# High Levels of Vitamin A in Plant-Based Diets for Gilthead Seabream (*Sparus aurata*) Juveniles, Effects on Growth, Skeletal Anomalies, Bone Molecular Markers, and Histological Morphology

**DOI:** 10.1155/2023/5788432

**Published:** 2023-12-14

**Authors:** David Dominguez, Daniel Montero, Maria Jesus Zamorano, Pedro L. Castro, Joseane da Silva, Ramon Fontanillas, Marisol Izquierdo

**Affiliations:** ^1^Aquaculture Research Group (GIA), University Institute of Sustainable Aquaculture and Marine Ecosystems (IU-ECOAQUA), University of Las Palmas de Gran Canaria, Road to Taliarte, 35200, Telde, Gran Canaria, Spain; ^2^Laboratory of Metabolism and Reproduction of Aquatic Organisms (LAMEROA), Department of Physiology, Institute of Biosciences, USP, Matão street, São Paulo, Brazil; ^3^Skretting Aquaculture Research Centre AS, PO Box 48, N-4001, Stavanger, Norway

## Abstract

Substitution of fish-based ingredients may alter the nutritional profile of the feeds, including the vitamin contents, ultimately leading to unbalanced vitamin supply. Vitamin A plays an essential role in epithelium preservation, cell differentiation, reproduction, and vision. It also intervenes in skeletogenesis through chondrocytes development. Therefore, low levels of vitamin A may cause poor growth and abnormal bone development among other symptoms. Besides, in gilthead seabream excess vitamin A altered bone structure and homeostasis, indicating that an upper level for vitamin A in feeds for this species must be defined. For this purpose, a practical plant-based diet (FM 10% and FO 6%) containing five increasing levels of vitamin A (24,000, 26,000, 27,000, 31,000, and 37,000 IU/kg) supplemented as retinyl acetate was formulated to identify the effects of high levels of vitamin A for gilthead seabream juveniles. The trial was conducted with 450 total fish distributed into 15 tanks, where each diet was tested in triplicates for 70 days. At the end of the trial, samples were taken for analyses of vitamin A—relevant markers. At the end of the trial the high levels of vitamin A supplementation did not cause a reduction in growth, whereas no significant effect was observed for the feed efficiency, specific growth rate, and feed convertion ratio. Although not significant, retinol content in liver showed a tendency to increase with the elevation of dietary vitamin A levels. Although minor, the highest level of vitamin A dietary content (37,000 IU/kg) caused a significant increase in caudal vertebrae partial fusion as well as caudal vertebrae malformations. Increasing dietary vitamin A was related to a reduction in the occurrence of microhemorrhages in the liver and a reduction in the presence of eosinophils associated to the pancreas. Overall, the results of the present study suggested that gilthead seabream juveniles fed a plant-based diet are able to tolerate very high levels of vitamin A supplementation when supplemented as retinyl acetate. Nevertheless, further supplementation should be avoided in order to reduce the prevalence of anomalies affecting the caudal vertebrae.

## 1. Introduction

Fishmeal and oil are rich in vitamin A [1], so the substitution of these ingredients by alternative ingredients will decrease the level of vitamin A in the feeds, ultimately leading to unbalanced vitamins supplies [2]. However, Hernandez and Hardy [3] indicate the lack of research assessing the effects of introducing plant-origin ingredients to replace fishmeal and fish oil in diets for fish, and how this will affect vitamin A nutrition.

Requirements for several vitamins have been established for species such as carps and salmonids [4], but adequate dietary levels for gilthead seabream (*Sparus aurata*) are yet unknown for most vitamins. In the recent years, some advances have been made in this regard, with studies on vitamin D [5, 6] and vitamin K [7] defining optimum levels for seabream. However, information regarding recommended vitamin A levels for this species remains unavailable.

Vitamin A preserves the epithelium, and is involved in cell growth and differentiation, reproduction, as well as in rhodopsin formation and regeneration, and maintaining resistance to infection [4, 8, 9]. This vitamin also takes part in the differentiation of osteoblasts and in the regulation of osteoclast activity, making in it a pivotal nutrient in skeletogenesis, and therefore, in reducing skeletal deformities [10]. Indeed, vitamin A regulates the expression of genes associated to bone development, including bone morphogenic protein, osteocalcin, or matrix Gla protein [11, 12]. Thus, a reduction in the level of dietary vitamin A can lead to lower growth and altered vision, hemorrhage, keratinization of the epithelia, and abnormal skeletal development [4, 8]. Therefore, several criteria have been employed to evaluate optimum vitamin A dietary levels in several fish species, including weight gain, vitamin A liver storage, fillet quality, vitamin A content in plasma, absence of skeletal deformities, and the lack of deficiency signs [3, 4, 8]. For instance, based on weight gain, optimum vitamin A levels range from as low as 0.3–0.6 mg/kg in channel catfish (*Ictalurus punctatus*; [13]) and 923 IU/kg (0.2769 mg/kg) in Amur sturgeon (*Acipenser schrenckii*; [14]), to as high as 31 mg/kg (1,03,000 IU/kg) in European seabass larvae (*Dicentrarchus labrax*; [15]) and 0.509–40.516 mg/kg (1697–135,053 IU/kg) in sunshine bass (*Morone chrysops × M. saxatilis*; [16]). Other authors describe the optimum levels for this nutrient on several fish species including 0.75 mg/kg (2,500 IU/kg) for rainbow trout (*Oncorhynchus mykiss*; [17]), 1.2–6.0 mg/kg (4,000–20,000 IU/kg) for common carp (*Cyrpinus carpio*; [18]), 0.10–0.12 g/kg for common carp (*Cyrpinus carpio* var. *communis*; [19]), 5.68 mg/kg (18,933 IU/kg) for yellowtail (*Seriola lalandi*; [20]), 0.93 mg/kg (3,100 IU/kg) for grouper (*Epinephelus tauvina*; [21]), 2.5 mg/kg (8,333 IU/kg) for Atlantic halibut (*Hippoglossus hippoglossus*; [22]), 2.7 mg/kg (9,000 IU/kg) for Japanese flounder (*Paralichtys olivaceus*; [23]), and 1.76–2.09 mg/kg (5,867–6,967 IU/kg) for hybrid tilapia (*Oreochromis niloticus × Oreochromis aureus*; [24]). Vitamin A can be found as retinol, retinal, retinoic acid, and retinyl esters [25, 26]. The aquaculture industry traditionally employs several sources of vitamin A for fish feeds, including retinol, retinyl acetate, retinyl palmitate, retinyl propionate, and carotenoids. When there is no information on the requirement for a given species or no requirements have been established, it is a common practice to use supplement recommendations for other species. This was already highlighted by Kaushik et al. [27] in their evaluation of NRC's 1993 recommendations for optimum vitamin levels in several fish species. As pointed out by the authors, despite these levels are sufficient to cover requirements for many fish species, exceptions were found, thus a safe margin is required when applying vitamin recommendations. Such was the case for vitamin requirements for salmonids, which were considered to be overestimations for other fish species, such as European seabass. This is highly relevant, since knowledge on salmonids' requirements is significantly more advanced than any other fish group [4], and fish feed producers often use these recommendations to elaborate feeds for other species, such as gilthead seabream. In fact, the effects of excess and deficiency of vitamin A causes similar clinical signs, and include keratinization of epithelia, altered growth, and skin lesions, but also hepatomegaly, splenomegaly, and bone anomalies [4, 8, 10, 28, 29]. Bone formation was also disturbed in European seabass larvae fed 1,000 mg of retinyl acetate/kg [15]. Other studies highlight its importance in skeletal development when defining the optimum vitamin A level for flatfish, species which suffer one of the most radical skeletal metamorphosis registered among fish [30]. Another sign of vitamin A excess is the alteration of the liver morphology to a pale and fragile liver, characteristics found in Japanese flounder fed diets with 25,000 IU/kg (7.5 mg/kg), suggesting a liver steatosis [23]. Vitamin A tends to accumulate mainly in the liver [18, 20, 22, 24], therefore, any morphological observations made in this tissue could be attributed to dietary vitamin A excess supplementation. Vitamin A can also influence the tissues' lipid composition [21], while other authors found dietary vitamin A affected stress-related parameters [16]. In gilthead seabream, excess vitamin A caused alterations in bone homeostasis and structure, increasing the deposition of osteocalcin and matrix Gla protein [12]. However, the latter study used only two levels of dietary vitamin A, a control containing 25,100 IU/kg (7.53 mg/kg), and an excess with 2,300,000 IU/kg (690 mg/kg), thus no recommendations could be made establishing upper levels of intake for this species.

Most vitamin A studies in fish have been conducted based on purified diets [16, 21, 24] or practical diets based on fish meal (FM) and oil (FO) [22], whereas, studies using alternative ingredients has been limited [9, 11]. However, the industry considers the substitution of FMFO a priority, and has been searching for alternatives for years, which forces to redefine optimum dietary levels in practical formulations [3]. Moreover, despite the negative effects observed when fish are fed an excess of this vitamin, studies addressing this issue in juvenile gilthead seabream are limited [12], and do not establish a safe upper limit for vitamin A supplementation. Thus, following the research by Fernández et al. [12], the aim of the present study was to evaluate the effects of high levels of vitamin A supplementation in plant-based feeds in growth, productive parameters, and health status of gilthead seabream juveniles to set a safe upper limit to the supplementation of vitamin A.

## 2. Materials and Methods

All the experimental conditions and sampling protocols have been approved by the Animal Welfare and Bioethical Committee from the University of Las Palmas de Gran Canaria.

### 2.1. Feeding Trial and Growth Performance

A practical plant-based diet (FM 10% and FO 6%) was formulated to contain a mixture of vegetable ingredients currently used for gilthead seabream in aquafeed, and supplemented to contain five increasing levels of vitamin A (24,000, 26,000, 27,000, 31,000, and 37,000 IU/kg; 7.2, 7.8, 8.1, 9.3, and 11.1 mg/kg) supplemented as retinyl acetate (Sigma Aldrich, Saint Louis, USA, 2,600,000–2,940,000 IU/g) (Table 1) based on the vitamin A content used by Fernández et al. [12] in their control diet. A common basal diet was used, thus the energy and nitrogen composition were equal. All the diets were designed to cover all known nutritional requirements for this species. Feeds were manufactured by extrusion process by Skretting Aquaculture Research Center AS (Stavanger, Norway). International Units (IU) have been used in an attempt to determine their equivalence to a known amount of retinol. In this sense, the current conversion ratio employed by the US Food and Drug Administration for 1 IU corresponds to 0.3 *µ*g of all-*trans*-retinol.

Four hundred and fifty gilthead seabream (*Sparus aurata*) juveniles, (20.4 ± 1.3 g mean BW) were selected and individually inspected to avoid the presence of external signs of skeletal anomalies. Fish were distributed into 15 tanks in triplicate groups per diet and fed until apparent satiation thrice daily for 70 days under a natural photoperiod (12 hr light). Water temperature (21.9 ± 0.2°C), oxygen (>5.8 ppm), and feed intake were monitored daily. Growth and productive parameters were monitored along the trial, for which fish were anesthetized with clove oil (Guinama S.L.U., Valencia, Spain) dissolved in the anesthesia tanks [31]. At the end of the 10-week trial all the fish were sampled for weight and length, and euthanized using ice after a 24-hour fasting period. Ten fish per tank were sampled, and their tissues stored frozen (−20°C) for both proximal composition and vitamin concentration; vertebrae from five fish per tank were flash-frozen using liquid nitrogen and kept at −80°C for further molecular analyses of bone markers; five fish per tank collected for histological evaluation and the samples stored at 10% buffered paraformaldehyde; the remaining 10 fish were stored frozen at −20°C for X-ray evaluation in order to conduct osteological assessment of skeletal anomalies.

### 2.2. Vitamin A Contents and Proximate Composition

Vitamin A (retinol) content was evaluated in feeds and liver by Eurofins Mas Control S.L. (Santa Cruz de Tenerife, Spain). Vitamin A was separated from the sample matrix by alkaline hydrolysis using ethanolic potassium hydroxide and extracted thrice with hexane:ethyl acetate (85 : 15 v/v). Determination was conducted using high performance liquid chromatography (HPLC) with ultraviolet/diode–array detection at 325 nm. All the reagents were suitable for HPLC analyses and contained a purity higher than 98%. Vitamin A content in feed was analyzed by Masterlab (Veerstraat 38, 5831 JN Boxmeer P.O. Box 220, 5830 AE Boxmeer, The Netherlands) using an HPLC-system [32].

Standard procedures were employed to evaluate the biochemical composition of diets and muscle [33]. Samples of diets and fillets were homogenized (T25 Digital Ultra-turraX, IKA®, Germany) before analysis. Crude lipid was extracted by chloroform:methanol [34] and ash by combustion in a muffle furnace (Carbolite, Sheffield, United Kingdom), at 600°C for 12 hr. Kjeldahl method was used to determine protein content (*N* × 6.25), whereas dry matter content was determined after drying the sample in an oven at 105°C until reaching constant weight.

### 2.3. Skeletal Anomalies

Radiographs were taken using a fixed X-ray apparatus (Bennett B-OTC, Bennett X-Ray Corp., Chicago, IL, USA) and a 35 × 43 cm digital film (Fujifilm FDR D-EVO (Fujifilm Corporation, Tokyo, Japan)). Fish were radiographed in groups of ten individuals. Radiographs were treated digitally (Onis 2.4, DigitalCore, Co.Ltd, Tokyo, Japan). Additionally, skeletal anomalies were classified according to Boglione et al. [35].

### 2.4. Gene Expression in Bone

#### 2.4.1. RNA Extraction

Vertebrae from fish fed the lowest, medium, and highest vitamin A contents were selected for studies of expression of bone-related genes. Sixty milligrams of vertebrae were used for total RNA extraction using TRI Reagent Solution (Life Technologies, Carlsbad, CA, USA) and RNeasy Mini Spin Columns (Qiagen, Hilden, Germany) were used for purification according to the manufacturer's instructions.

#### 2.4.2. Reverse Transcription

One microgram total RNA from each experimental sample was reversed transcripted with iScript cDNA synthesis kit (Bio-Rad Laboratories, Hercules, CA, USA) as indicated by the manufacturer's instructions with slight modifications. Briefly, 1 *µ*g total RNA and nuclease-free water to a final volume of 15 *µ*l. Afterward, the mix was heated at 65°C for 10 min and cooled in ice. 1 *µ*l of iScript reverse transcriptase and 4 *µ*l of 5 × iScript reaction mix were then added, reaching a final volume of 20 *µ*l. The reaction mix was incubated for 5 min at 25°C, 30 min at 42°C, and then 5 min at 85°C to inactivate reverse transcriptase. The reverse transcription reactions were diluted 1 : 10 for gene quantification.

#### 2.4.3. Quantitative Polymerase Chain Reaction (PCR)

The nucleotide sequences of primers used in this study are reported in Table 2. Real-time PCR was used for gene expression quantification using the iQ5 Multicolor Real-Time PCR detection system (Bio-Rad Laboratories, Hercules, CA, USA). IQ^TM^ SYB® Green Supermix (Bio-Rad Laboratories, Hercules, CA, USA) and a total of 2 *µ*l of diluted cDNA were used for the reaction. Each sample, housekeeping, and target gene were analyzed in duplicates, and using in a final reaction volume of 20 *µ*l. Beta actin (*bact*) and elongation factor 1-alpha (*ef1α*) were used as housekeeping genes to normalize the expression of the target genes in vertebrae. The PCR conditions were as follows: 95°C for 3 min and 30 s, followed by 40 cycles of 95°C for 15 s, 58.1°C for 30 s, and 72°C for 30 s; 95°C for 1 min, and a final denaturation step from 58 to 95°C for 10 s. The 2^−*ΔΔ*Ct^ method [36] was applied to analyze the relative changes in gene expression.

### 2.5. Histological Studies

Samples were further segmented to allow a better penetration of the alcohol and introduced in histology cassettes. Dehydration of the samples was carried out using a Histokinette 2000 (Leica, Nussloch, Germany) with gradually increasing alcohol grades beginning with 70° and ending with 100°, being the last two steps xylene and paraffin. Once the paraffin block was obtained it was sliced at a thickness of 3 *µ*m using a Leica RM 2135 microtome (Leica, Nussloch, Germany) and fixed to a slide including as much parts of the tissue as possible. Samples were then stained with hematoxylin—eosin staining Martoja and Martoja-Pierson [37] for optical evaluation. Once the preparations were ready they were subjected to optical analysis in search for signs of liver and pancreas damage such as fat accumulation, congestion, signs of inflammation, and presence of eosinophils, hemorrhages, and bile duct obstruction, and analyzed by pair evaluators in a 0–3 scale, where 0 was absence of observation and 3 presence in most of the tissue.

### 2.6. Statistics

All data presented in this manuscript were statistically analyzed using SPSS v21 (IBM Corp., Chicago, IL, USA) and means ± SD were calculated for every parameter measured. One-sample Kolmogorov–Smirnov test was employed to evaluate the normality of the data. One-way analysis of variance (ANOVA) was then used to determine the effects of the different diets for normally distributed data. Data were tested for homogeneity of variances and post hoc analysis was carried out using Tukey test if variances were homogeneous or Games–Howell test whenever variances were different. Logarithmic or arcsin transformation was carried out and the nonparametric tests of Kruskal–Wallis test was used in those cases where data did not follow a normal distribution. Quadratic and lineal regressions and broken line analyses were conducted where necessary. The error analysis presented in these figures revealed that the ratio lack-of-fit to pure error variances gives evidence to support the adequacy of the models described by the equations presented throughout the manuscript. Significant differences were considered for *p* < 0.05.

## 3. Results

### 3.1. Feeding Trial and Growth Performance

All the diets were well accepted by the fish and no mortalities were registered. At the end of the feeding trial, all fish had at least doubled their weight. The lowest weight gain was registered in fish fed the diet containing 26,000 IU, followed by the diet containing 24,000 IU, whereas fish fed the rest of the diets presented a significantly higher body weight compared to those fed 26,000 IU (Table 3). There were no statistical differences in feed efficiency, specific growth rate, and feed convertion ratio.

### 3.2. Vitamin A Contents and Proximate Composition

There were no significant (*p* > 0.05) differences in retinol content in liver, but the diet with the highest supplementation showed a 21% increase compared to the lowest supplemented diet (120.3 vs. 98.9 mg/kg, respectively, Table 4). Biochemical composition in terms of muscle lipids, protein, and ash of seabream juveniles at the end of the trial was not significantly (*p* > 0.05) affected by dietary vitamin A supplementation, and the results obtained for muscle ash and protein constant remained almost identical between the treatments (Table 4).

### 3.3. Skeletal anomalies

**Figure 1:**
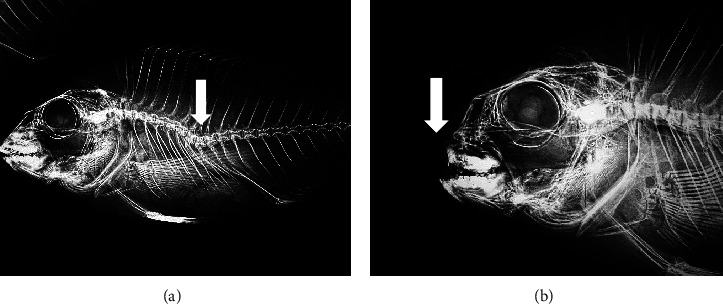
Gilthead seabream juveniles showing different types of skeletal anomalies. (a) Prehaemal lordosis. (b) Maxillary and/or premaxillary anomaly.

**Figure 2:**
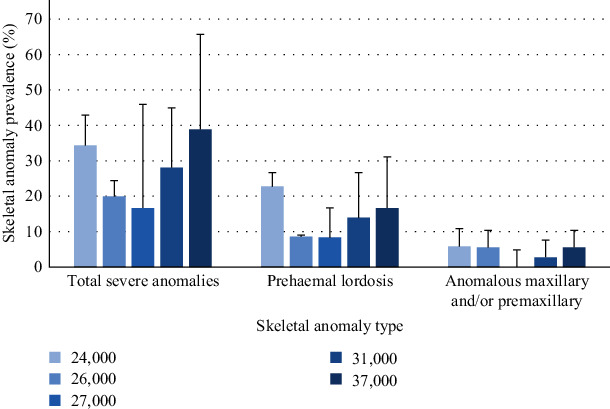
Prevalence of severe anomalies; prehaemal lordosis; and anomalous maxillary and/or premaxillary (%) in seabream fed increasing levels of dietary vitamin A for 70 days.

**Figure 3:**
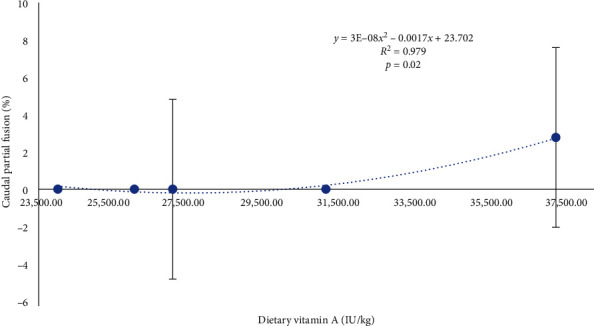
Prevalence of caudal vertebrae partial fusion (%) in seabream fed increasing levels of dietary vitamin A for 70 days.

At the end of the trial, skeletal anomalies were predominantly found in the anterior region including cranium and, prehaemal vertebrae (Figure 1), whereas other anomalies such as prehaemal fusions, prehaemal anomalies, haemal lordosis, or caudal anomalies presented a low prevalence (2.3% ± 1.71%, mean ± SD). There was a high variation on the incidence in all the types of anomalies studied, and no statistically significant differences were found between experimental treatment (or diets). Results are somehow difficult to interpret. For instance, in seabream fed the diet containing the lowest vitamin A level (24,000 IU/kg) the incidences of frequency of anomalies and prehemal lordosis were 2 and 2.7 times higher than in fish fed 27,000 IU/kg of vitamin A (Figure 2). However, seabream fed the highest vitamin A levels (37,000 IU/kg) also showed 2 and 2.3 times higher incidence of frequency of anomalies and prehemal lordosis than fish fed 27,000 IU/kg vitamin A. Additionally, no signs of maxillary and/or premaxillary anomalies were found in fish fed 27,000 IU/kg, whereas the incidence of this cranial anomaly reached an average of 5.8% of the fish fed 24,000 or 37,000 IU/kg of vitamin A. However, no significant differences were registered (*p* > 0.05) among the mean values per diet, due to the large standard deviations. Nevertheless, caudal vertebrae partial fusion was significantly increased by the highest inclusion of dietary vitamin A (*y* = 3E−08*x*^2^ − 0.0017*x* + 23.702; *p*=0.02, *R*^2^ = 0.979, Figure 3), as well as caudal vertebrae malformations (*y* = 0.2358*x*– 2.673; *p*=0.05).

### 3.4. Gene Expression in Bone

No significant (*p* > 0.05) differences were found among the mean values for the expression of bone molecular markers (Table 5). However, expression of *bmp2* was negatively related to the retinol contents in liver (*R*^2^ = 0.99; *p*=0.015). Thus, fish fed 27,000 IU/kg vitamin A showed a *bmp2* expression that was 143% higher than that of fish fed 37,000 IU/kg vitamin A (Table 5). Besides, *alp* expression was highly related to the dietary vitamin A levels (*R*^2^ = 0.99; *p*=0.028), while presenting a tendency with the prevalence of caudal anomalies (*R*^2^ = 0.935; *p*=0.164) or caudal partial fusion (*R*^2^ = 0.931; *p*=0.169).

### 3.5. Histological Studies

**Figure 4:**
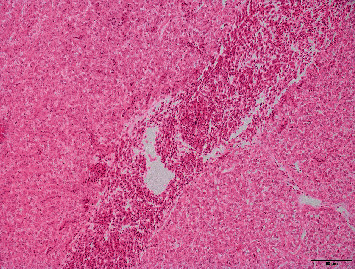
Widespread presence of microhemorrhages in the liver of gilthead seabream juveniles from the present trial.

**Figure 5:**
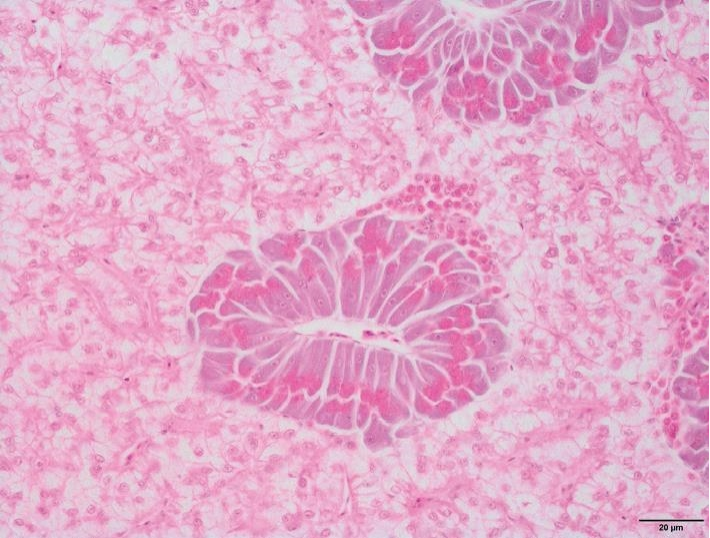
Presence of eosinophils in the pancreas of gilthead from the present trial.

**Figure 6:**
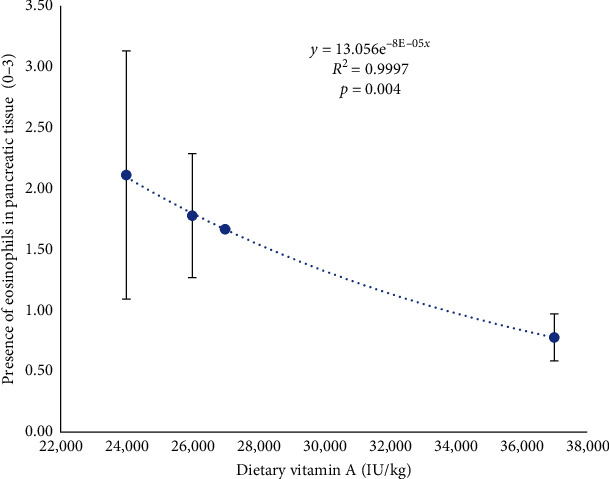
Presence of eosinophils associated to the pancreas in seabream fed increasing levels of dietary vitamin A for 70 days.

From a histological point of view, liver and pancreas morphology were very similar among fish fed the different dietary vitamin A levels. In this sense, liver steatosis (2.87 ± 0.10), congestion (2.15 ± 0.18), inflammatory infiltrate (0.72 ± 0.46); and pancreas hemorrhages (1.86 ± 0.19), fat (0.86 ± 0.43), presence of melanomacrophages (0.94 ± 0.06), and inflammatory infiltrate (0.32 ± 0.17) were not statistically different among the treatments. However, lineal models proved that an increase in dietary vitamin A was slightly related to a reduction in the occurrence of microhemorrhages in the liver (*R*^2^ = 0.95; *p*=0.150; Figure 4) and a significant reduction in the presence of eosinophils associated to the pancreas (*R*^2^ = 0.99; *p*=0.004; Figures 5 and 6).

## 4. Discussion

Despite the importance of correct dietary levels of vitamin A to promote fish growth, epithelial integrity, or bone condition, vitamin A requirements for gilthead seabream have not been yet determined. Moreover, diets high in fish-derived ingredients contain retinyl esters or vitamin A precursors from xanthophylls, including astaxanthin, as sources of vitamin A, whereas carotenoids are more commonly found in plant-based diets, being beta-carotene the main precursor due to the low activity of xanthophylls. Given the present replacement of FM and FO by plant sources in diets for gilthead seabream, it is necessary to understand the effects of dietary vitamin A levels in this type of diets. The present study has shown that high levels of vitamin A in the form of retinyl acetate, do not cause a reduction in seabream growth in terms of final body weight. Growth is commonly used to define vitamin A requirements in aquaculture species [15, 17, 18, 24], together with the liver storage of vitamin A [18, 20, 22–24]. In higher vertebrates, up to 90% of vitamin A is stored in the liver [38]. For instance, in juvenile hybrid tilapia (*Oreochromis niloticus* × *O. aureus*) increase in dietary vitamin A, supplemented as retinyl acetate, increased vitamin A contents in liver [24], and a similar result was obtained in Atlantic salmon postsmolts [39]. Nevertheless, in the present study, retinol contents in liver did not increase with dietary levels of vitamin A, meaning that either the level intervals were too small to show a significant effect on the accumulation of retinol in the liver, or that the deposition of vitamin A was not done in the form of retinol, but in other forms.

The important role of vitamin A in bone development is complex and partly mediated by the regulation of osteoblast and osteoclast activities. Therefore, deficiency in vitamin A has been associated to defective remodeling of intramembranous bone [40]. Besides, vitamin A is also involved in mucopolysaccharides synthesis that are components of cartilage and bones. Thus, vitamin A deficiencies may lead to disorganized bone growth and subsequent anomalies. For instance, vitamin A deficiency in sea bass impairs the formation of pelvic fins [41]. In agreement, in the present study in seabream fed the lowest vitamin A levels (24,000 IU/kg) the incidence of anomalies was at least double than in fish fed 27,000 IU/kg of vitamin A. Besides, no anomalous maxillary and/or premaxillary were found in fish fed 27,000 IU/kg, whereas the incidence of this cranial anomaly reached an average of 5.8% of the fish fed 24,000/IU kg of vitamin A. These results are in agreement with the role of vitamin A on function of chondrocytes [10], which are required for the formation of endochondral bones such as the seabream cranial bones.

Despite vitamin A is necessary for bone formation, the excess of retinoic acid may promote bone resorption, reduce bone formation, and lead to bone anomalies. This effect has been observed in several fish species. Atlantic salmon postsmolts fed diets containing over 360 mg/kg retinol (as retinyl acetate), presented an increase in the craniofacial and spinal deformities [39]; whereas in gilthead seabream, hypervitaminosis A caused skeletal malformations by affecting both compact and trabecular bone layers and their calcification pattern in vertebrae [12]. In agreement, the present trial showed that the incidence of skeletal anomalies in seabream fed the highest vitamin A levels (37,000 IU/kg) also doubled that of fish fed 27,000 IU/kg. Moreover, increase of dietary vitamin A was significantly related to the increase in caudal vertebrae partial fusion. Previous studies specifically designed to determine the effect of hypervitaminosis A in gilthead seabream by feeding the juveniles with 2,300,000 IU/kg vitamin A have shown strong evidences of skeletal malformation even after 3 months of feeding [12]. Retinoids, particularly all-*trans*-retinoic acid, induce osteoblast differentiation, but require bone morphogenetic proteins to stimulate both osteoblast differentiation and bone formation [40]. Thus, retinoic acid interacts with *bmp2* to induce osteoblastic differentiation [42]. Besides, *bmp2* mediates different retinoid-induced effects in cell differentiation processes [43], including skeletogenesis [44]. In agreement, in the present study, seabream fed 27,000 IU/kg vitamin A showed a 2.4 higher expression of *bmp2* in vertebrae than fish fed 37,000 IU/kg vitamin A, and the expression of this gene was inversely correlated to the storage of retinol in liver and the frequency of anomalies. Moreover, *bmp2* regulates the expression of cellular retinoic acid binding proteins (*crabp1*) [45], which in turns mediates the vitamin A-induced skeletal anomalies in Senegalese sole (*Solea senegalensis*, [46]). Bone health and homeostasis depends on the bone development and remodeling processes that are controlled by both retinoic acid and *bmp2*, among other signaling elements. In higher vertebrates increased retinoic acid levels have been associated to increased *alp* activity. For instance, raised levels of retinoic acid increased the *alp* activity in mice bone marrow stromal cells, but inhibited *oc* expression and mineralization, in a process not mediated by *runx2* [42]. In the present trial, there was a nonsignificant (*p*=0.097) lineal increase in the expression of *alp* with increasing level of vitamin A supplementation, which agrees with the observations made in mice. However, the *oc* expression is not significantly affected by dietary vitamin A, which might suggest there is a difference in the molecular mechanisms between these two species.

Vitamin A is metabolized in the liver after its absorption at the intestine, where it is incorporated into the hepatocytes [30]. Vitamin A excess in Japanese flounder caused “pale and fragile” livers [23], symptoms that indicate the presence of liver steatosis. Gilthead seabream liver and pancreatic tissues are only distinguishable at a microscopic level, since the tissues are found in the hepatopancreas. Thus, the present study evaluated these tissues jointly in order to discern between steatosis and other symptoms of liver and pancreatic damage. Nevertheless, the only significant effects of vitamin A on these tissues were an increased presence of inflammatory infiltrate, indicative of hepatitis, only at 27,000 IU/kg vitamin A, meaning that the mechanisms observed in Japanese flounder differs significantly from the one observed in seabream, and suggesting that this species might be more resistant to vitamin A toxicity in the liver. Another observation made in the present study was reduction in the presence of eosinophils in the pancreas with increasing vitamin A level. As far as the authors are aware of, this is the first time this observation has been made in fish. In fact, the only observations relating vitamin A with alterations in the eosinophil count have been made in rats and mice and related to lung diseases (granulomatosis and asthma). The first study saw a reduction in the count of eosinophils in the lungs of rats administered increasing levels of vitamin A intravenously [47], which, according to the authors, was caused by a suppression of tumor necrosis factor alpha (TNF-*α*) and eotaxin production and nuclear factor kappa-light-chain-enhancer of activated B cells activation which reduced the lung inflammatory response. On the second study, the authors saw an increase in the bronchoalveolar lavage count of eosinophils with increasing dietary vitamin A in mice [48] caused by, what the authors argued to be, an increase in the development and trafficking of granulocyte from the bone marrow to the lung caused by vitamin A. As can be observed, the mechanisms underlying the effects of vitamin A in the activation of this cell line are not fully understood in more stablished laboratory animals such as mice and rats, so it is out of the scope of the present article to address them in depth in a relatively new laboratory animal as the seabream. Nevertheless, the present study does prove that there must be some similarities worth studying in the future between fish and mammal immune system and its relation with vitamin A. Overall, the results of the present study suggested that gilthead seabream juveniles are able to tolerate high levels of vitamin A supplementation, showing that there were no negative effects of the vitamin supplementation in terms of final body weight or weight gain, whereas the highest level of supplementation caused a slight increase in relation to skeletal deformities. As for histology, the highest vitamin A dose reduced liver eosinophil infiltration and liver microhemorrhages in gilthead seabream. It is important to note that the high levels of vitamin A used in this study are comparable to those employed in another study with European seabass, where the basal diet already contained 40,000 IU/kg of all-*trans*-retinol [15], and in gilthead seabream where the basal diet contained 25,100 IU/kg [12], and these levels where already very high when compared to trials conducted in other species. For instance, trials conducted in Atlantic halibut with practical diets, presented relatively low levels of vitamin A (0.25 IU/kg) in the basal diet [22] compared to the present trial. This dietary level of vitamin A is higher than that recommended for several species including grouper (3,101 IU/kg, [21]), sunshine bass (1,700–135,000 IU/kg, [16]), Japanese flounder (9,000 IU/kg, [23]), or hybrid tilapia (5,850–5,670 IU/kg, [24]), when purified or semipurified diets were used. It would therefore, be of interest to conduct further trials with no vitamin A supplementation for gilthead seabream juveniles, as well as to study how the presence of other nutrients or ingredients may affect vitamin A availability.

## Figures and Tables

**Table 1:** Ingredients and analyzed vitamin A content of the diets supplemented with retinyl acetate fed to gilthead seabream for 70 days.

Ingredient						g/kg
Corn gluten^a^						150
Wheat gluten^b^						217.3
Soya bean concentrate^b^						230
Faba beans^b^						50
Fish meal, Scandinavian^c^						100
Wheat^b^						116.0
Rapeseed oil^b^						31.1
Linseed oil^d^						8.2
Fish oil, South American^b^						60
Palm oil^e^						16.3
Premixes^f^						21.1

Analyzed content	VA24	VA26	VA27	VA31	VA37	
Vitamin A (IU/kg)	24,000	26,000	27,000	31,000	37,000	
Vitamin A (mg/kg)	7.2	7.8	8.1	9.3	11.1	

Proximal compostion (fresh weight) (%): lipids: 17.7, proteins: 50.9, ashes: 4.0. ^a^Cargill B.V., Amsterdam, The Netherlands. ^b^Skretting, Stavanger, Norway. ^c^Norsildmel, Bergen, Norway. ^d^Linagro, Lichtervelde, Belgium. ^e^AAK AB, Karlshamn, Sweden. ^f^Trouw Nutrition, Boxmeer, The Netherlands. Proprietary composition Skretting ARC, vitamin, and mineral supplementation as estimated to cover all known requirements for this species [4] except for vitamin A that was added separately. Micronutrient premix, inositol, monocalcium phosphate, l-lysine, Dl-methionine, minerals, and other vitamins.

**Table 2:** Sequences of primers used for gene expression analyses.

Gene	Nucleotide sequence (5′–3′)	Accession number
Beta-actin (*bact*)	F: TCTGTCTGGATCGGAGGCTC	X89920
	R: AAGCATTTGCGGTGGACG	

Elongation factor 1-*α* (*ef1α*)	F: CATGGTTGTGGAGCCCTTCT	AF184170
	R: TCCTGCACGACCATTCATTTC	

Alkaline phosphatase (*alp*)	F: AGAACGCCCTGACGCTGCAA	AY266359
	R: TTCAGTATACGAGCAGCCGTCAC	

Runx-related transcription factor 2 (*runx2*)	F: GCCTGTCGCCTTTAAGGTGGTTGC	AJ619023
	R: TCGTCGTTGCCCGCCATAGCTG	

Osteocalcin (*oc*)	F: GGCAGCCATCTGTCTGACTT	AF048703.1
	R: GGTCCGTAGTAGGCCGTGTA	

Bone morphogenic protein 2 (*bmp2*)	F: GTGGCTTCCATCGTATCAACATTTT	JF261172.1
	R: GCTCCCCGCCATGAGT	

**Table 3:** Growth parameters of gilthead seabream fed increasing contents of vitamin A for 70 days.

Vitamin A IU/kg	24,000	26,000	27,000	31,000	37,000
Initial weight (g)	20.1 ± 0.1	20.4 ± 0.1	20.3 ± 0.2	20.3 ± 0.0	20.4 ± 0.4
WG	134.3 ± 4.9^ab^	125.2 ± 5.4^a^	138.4 ± 41.4^b^	141.8 ± 11.8^b^	146.7 ± 15.1^b^
FE	0.91 ± 0.02	0.83 ± 0.07	0.73 ± 0.32	0.86 ± 0.05	0.86 ± 0.10
SGR	1.25 ± 0.03	1.19 ± 0.04	1.26 ± 0.27	1.30 ± 0.07	1.33 ± 0.09
FCR	1.10 ± 0.03	1.21 ± 0.11	1.65 ± 0.97	1.17 ± 0.07	1.17 ± 0.14

Different letters in the same row indicate significant differences, *p* < 0.05, *n* = 3. FCR, feed convertion ratio; FE, feed efficiency; SGR, specific growth rate; WG, weight gain.

**Table 4:** Liver retinol contents and muscle composition (dry weight) (%) of gilthead seabream fed increasing contents of vitamin A for 70 days.

Vitamin A (IU/kg)	24,000	26,000	27,000	31,000	37,000
Liver retinol (mg/kg)	98.9 ± 19.8	113.1 ± 22.6	87.5 ± 17.5	112.0 ± 22.4	120.3 ± 24.1
Muscle lipids (d.w.) (%)	13.9 ± 1.0	14.1 ± 1.3	14.7 ± 1.8	15.4 ± 0.7	11.9 ± 0.6
Muscle ash (d.w.) (%)	1.5 ± 0.1	1.6 ± 0.0	1.6 ± 0.0	1.5 ± 0.0	1.5 ± 0.0
Muscle protein (d.w.) (%)	21.0 ± 0.3	20.9 ± 0.2	20.5 ± 0.5	21.0 ± 0.1	20.6 ± 0.1

**Table 5:** Vertebra gene expression analyses of gilthead seabream fed increasing levels of dietary vitamin A for 70 days.

Vitamin A (IU/kg)	24,000	27,000	37,000	*p* Value
*runx2*	1.01 ± 0.15	1.36 ± 0.20	1.33 ± 0.21	0.113**q**
*bmp2*	1.00 ± 0.07	1.24 ± 1.81	0.51 ± 0.39	0.469**l**
*alp*	1.01 ± 0.19	1.09 ± 0.17	1.29 ± 0.23	0.097**l**
*oc*	1.13 ± 0.68	0.62 ± 0.45	0.97 ± 0.30	0.49**q**

Different letters in the same row indicate if the data adjusted to a linear (**l**) or quadratic (**q**) model. Nevertheless, none of the parameters studied presented statistically significant values.

## Data Availability

The majority of data used to support the findings of this study are included within the article. The histological analysis data used to support the findings of this study are available from the corresponding author upon request.
